# The Impulsivity-Compulsivity Spectrum: Understanding Brain Mechanisms and Clinical Implications

**DOI:** 10.1192/j.eurpsy.2024.1303

**Published:** 2024-08-27

**Authors:** C. Pinheiro Ramos, C. Baptista, J. Nogueira, S. Mendes, A. Gamito

**Affiliations:** Setúbal Hospital Center, Setúbal, Portugal

## Abstract

**Introduction:**

Impulsivity and compulsivity are natural behaviors controlled by brain mechanisms that are essential for survival in all species. Understanding these brain mechanisms may lead to targeted treatment strategies for these symptom domains when impulsivity and compulsivity become dysfunctional.

Pathological impulsivity and compulsivity characterise a broad range of mental disorders.

**Objectives:**

This study aimed to synthesise the latest evidence about the conceptualization of the impulsivity-compulsivity spectrum.

**Methods:**

A review was conducted, drawing on reputable sources (PubMed and Web of Science databases).

**Results:**

The concept of impulsivity can be defined as a predisposition toward rapid, unplanned reactions to internal or external stimuli without regard to the negative consequences of these reactions to the impulsive individual or to others. However, impulsivity is not always unplanned. Impulsive behaviours can be conceptualised as the core symptoms of a broad range of psychiatric disorders.

In contrast, compulsivity refers to repetitive behaviours that are performed according to certain rules or in a stereotypical fashion. Compulsivity is a tendency to repeat the same, often purposeless acts, which are sometimes associated with undesirable consequences.

Impulsivity and compulsivity may be viewed as diametrically opposed, or alternatively, as similar, in that each implies a dysfunction of impulse control. Each involves alterations within a wide range of neural processes, including attention, perception, and coordination of motor or cognitive responses.

**Conclusions:**

The neurobiology of impulsivity and compulsivity may involve inhibitory neurotransmitters, excitatory neurotransmitters, the prefrontal cortex, and/or limbic dysfunction.

Impulsive and compulsive features may present at the same time or at different times during the same illness. Although both compulsive and impulsive disorders may be related to prefrontal cortex dysfunction, compulsive disorders would be related to hyperactivity and impulsive disorders to hypoactivity of the prefrontal cortex. Compulsiveness appears to be associated with increased frontal lobe activity, while impulsiveness may be associated with reduced frontal lobe activity.

**Disclosure of Interest:**

None Declared

